# Nonimmunoglobulin Crystal-Storing Histiocytosis (CSH): Case Report and Literature Review

**DOI:** 10.1155/2020/8856411

**Published:** 2020-10-19

**Authors:** Manuel Beltran, Sharad Khurana, Yennifer Gil, Jason T. Lewis, Rohit Kumar, James M. Foran

**Affiliations:** ^1^Division of General Internal Medicine, Mayo Clinic, Jacksonville, FL, USA; ^2^Division of Hematology & Oncology, University of Arizona Cancer Center, Tucson, AZ, USA; ^3^Department of Anesthesia and Preoperative Medicine, Mayo Clinic, Jacksonville, FL, USA; ^4^Department of Pathology, Mayo Clinic, Jacksonville, FL, USA; ^5^Division of Medical Oncology and Hematology, University of Louisville, Louisville, KY, USA; ^6^Department of Hematology & Oncology, Mayo Clinic, Jacksonville, FL, USA

## Abstract

Crystal-storing histiocytosis (CSH) is an uncommon condition in which histiocytes accumulate a crystalline matter within their cytoplasm. Generally, those crystals are composed of either monoclonal or polyclonal immunoglobulin chains, which have a strong association with an underlying lymphoproliferative or plasma cell disorder (LP-PCD). Rarely, CSH has been reported as local or generalized manifestation of a variety of benign disorders. These cases are associated with crystals composed of nonimmunoglobulin substances. We are reporting an exceptional case of a local colonic CSH with Charcot–Leyden crystals. This patient underwent a screening colonoscopy that detected some polyps. The biopsy reported tubular adenomas, with a markedly dense, transmural inflammatory infiltrates, which were predominantly composed of eosinophils and crystal-storing histiocytes containing Charcot–Leyden crystals. The patient had a negative workup for LP-PCD and autoimmune conditions, including a normal skeletal survey and bone marrow aspirate/biopsy. The only positive laboratory workup was an elevated absolute eosinophil count and a positive IgG anti-*Strongyloides* antibody. Giving those findings, this parasitic infection is the most likely etiology of the CSH in our patient. Although there was an initial negative evaluation for LP-PCD, close monitoring of patients with either immunoglobulin or nonimmunoglobulin CSH is recommended.

## 1. Introduction

Crystal-storing histiocytosis (CSH) is a unique entity presenting as sheets of histiocytes with accumulation of crystalline matter within the cytoplasm. It can present as either a localized or generalized disease and has a variable outcome depending on the underlying CSH etiology [1]. Most often, the crystals are composed of monoclonal or polyclonal immunoglobulin light or heavy chains and have a strong association with an underlying lymphoproliferative or plasma cell disorder (LP-PCD) [1–3]. Rarely, the crystals are composed of nonimmunoglobulin substances such as clofazimine, cystine, silica, or Charcot–Leyden crystals and are often seen in association with a variety of benign disorders [1]. Literature on nonimmunoglobulin CSH is scarce. We discuss here a case of presentation with a colonic-only nonimmunoglobulin CSH with Charcot–Leyden crystals, along with a review of literature on this rare disease.

## 2. Case Description

**Figure 1:**
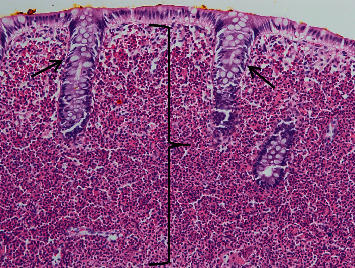
Medium power H&E of the colonic mucosa demonstrating a diffuse expansion of the lamina propria by eosinophils (denoted by black bracket). There is displacement of the colonic crypts (black arrows). No other inflammatory cell types are seen.

**Figure 2:**
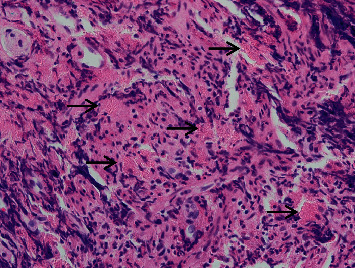
High power H&E showing aggregates of histiocytes within the muscularis mucosa and superficial submucosa. The cytoplasm of the histiocytes contains eosinophilic Charcot–Leyden crystals which are present in the form of fine, granular, and needle-shaped deposits within the macrophage cytoplasm (black arrows).

A 55-year-old man with a history of hypertension, hyperlipidemia, gout, recurrent *H. pylori* gastritis, gastroesophageal reflux with reflux esophagitis and esophageal stricture underwent a screening colonoscopy which showed polyps along with white nodular submucosa in the proximal ascending colon. Biopsy confirmed tubular adenomas, with a markedly dense, transmural inflammatory infiltrates predominantly composed of eosinophils and crystal-storing histiocytes containing Charcot–Leyden crystals (Figures 1 and 2). Besides the recurrent dyspeptic symptoms and dysphagia, he denied any other gastrointestinal symptoms such as cramping, diarrhea, or constipation. Review of system was negative for any fatigue, recurrent infections, bony pain, or easy bleeding/bruising. Laboratory workup showed an elevated absolute eosinophil count of 500 cells/**μ**l and a positive IgG anti-*Strongyloides* antibody. Serum evaluation for a monoclonal protein, serum free light chains, lactate dehydrogenase (LDH), and beta-2 microglobulin was normal. Tryptase levels and *CHIC2*, *PDGFRA*, and *FIP1L1* gene regions were normal as well. A negative autoimmune workup was noted. Skeletal survey showed no lytic or blastic bony lesions, and CT scan of the chest, abdomen, and pelvis showed no lymphadenopathy or hepatosplenomegaly. Bone marrow aspirate/biopsy did not show any evidence of an underlying plasma cell disorder or any mast cell disorder. The patient was treated with ivermectin for *Strongyloides stercoralis* infection and continued to follow-up with gastroenterology for his antibiotic and pantoprazole-treated *H. pylori* gastritis and esophagitis. He continued to follow closely with hematology for monitoring of development of any LP-PCD in future.

## 3. Discussion

Around 90% cases of crystal-storing histiocytosis (CSH) are associated with an underlying LP-PCD such as multiple myeloma (MM), lymphoplasmacytic lymphoma (LPL), and monoclonal gammopathy of undetermined significance (MGUS) [1, 2]. Overproduction of kappa light chain protein, abnormalities in specific stored paraprotein sequences, and resistance to intralysosomal degradation have been proposed as possible mechanisms of accumulation and crystallization of immunoglobulins in the cytoplasm of histiocytes [3, 4].

Approximately 8.8% of CSH cases are seen in association with a variety of benign disorders. Conditions with hyperactivated immune system such as rheumatoid arthritis, Fanconi syndrome, Crohn's disease, eosinophilic colitis, and mastocytosis; infections like *H. pylori*; and drugs like clozafamine have been reported in patients with CSH without an underlying LP-PCD [2, 3]. In these cases, materials different from immunoglobulins have been described, including clofazimine-induced CSH, Charcot–Leyden crystal-associated CSH, and CSH associated with hereditary cystinosis [2] (Table 1).

Besides the composition of the crystals, CSH can be classified as localized CSH (L-CSH), involving only one organ or site such as lung, pleura, stomach, kidney, bone marrow, thyroid, thymus, and parotid gland [3, 4], and generalized CSH (G-CSH), involving two or more distant organs or sites. [3]. According to Dogan et al., only 8% of localized CSH cases have been detected in the gastrointestinal tract. In fact, just 3 cases of colonic CSH have been described in the literature so far [3, 8], seen in association with eosinophilic colitis, MGUS, and lymphoma. We present here, colonic-only Charcot–Leyden crystals (CLC) CSH, possibly related to the patient's history of strongyloidiasis with associated peripheral eosinophilia.

Charcot–Leyden crystals have hexagonal and bipyramidal forms [11]. They are compounds of galectin-10 (Gal-10), a member of the family of S-type lectin proteins [12]. Gal-10 is a major constituent of eosinophils, estimated to be around 7–10% of total cellular proteins. A higher percentage is usually a sign of eosinophilic inflammation [12]. This protein is insoluble at neutral pH, remarkably resistant to various enzymes, and exhibits a tendency to form noncovalent aggregates [12]. Activated eosinophils trigger a nonapoptotic extracellular trap cell death called *ETosis*, where Gal-10 is homogeneously redistributed in the cytoplasm, followed by intracellular CLC formation. Subsequently, the eosinophil plasma membrane ruptures, releasing chromatin and vesicles together with CLC [12]. Additionally, pathogens and parasites may also induce eosinophilic apoptosis triggering a massive eosinophilic release of Gal-10, thereby resulting in extracellular CLC formation [11, 12]. It has been demonstrated both in vitro and in vivo that macrophages/histiocytes are able to engulf CLC and Gal-10 to form additional CLCs in the phagosomes of macrophages, leading to CLC CSH [11, 12].

Strongyloidiasis is a disease caused by a nematode *Strongyloides stercoralis*. Clinical manifestations can range from asymptomatic eosinophilia to gastrointestinal symptoms, such as upper abdominal pain, diarrhea, anorexia, nausea, and vomiting. In addition, patients can develop cutaneous or pulmonary symptoms [13]. *Strongyloides* causes direct eosinophilic infiltrate in the colonic mucosa [14], and as a result, CLC deposits could be expected even in the absence of colorectal symptoms, as was seen in our patient. *H. pylori* have been described as a cause of CSH in the stomach [15]. Therefore, it could potentially be a differential etiology for our patients with colonic CSH. However, the colonic location of CSH, the presence of eosinophilic infiltrate in the colonic polyp biopsy, peripheral eosinophilia, and the positive IgG anti-*Strongyloides* antibody make *H. pylori* an unlikely etiology of our patients with CSH [14].

CSH has been reported as a first manifestation or a subclinical stage of underlying neoplasms such as thymic lymphoma, multiple myeloma, or lymphoreticular malignancy [1, 15]. As such, an initial negative evaluation for an LP-PCD does not obviate the need for a close follow-up in patients diagnosed with either immunoglobulin or nonimmunoglobulin CSH.

In conclusion, CSH is an uncommon phenomenon of aggregation of crystals within the histiocytic cytoplasm, which are usually made up of immunoglobulins. Nonimmunoglobulin CSH is extremely rare and is usually associated with benign conditions. We report here a Charcot–Leyden colonic-only CSH associated with *Strongyloides* infection in the absence of LP-PCD.

## Figures and Tables

**Table 1:** Nonimmunoglobulin CSH reported in literature.

Source	Sex/age	Patient history/underlying disease	Organ(s) involved by CSH	Type of crystal	Symptoms/indication for workup	Disease status at last follow-up
Sukpanichnant [5]	M/32 y	Leprosy	SI and LN	Clofazimine crystals	Chronic abdominal pain	Died 2 months after diagnosis; had an acute onset of dyspnea
Gebrail [6]	M/23 y	Hereditary cystinosis	Cornea, conjunctiva, BM	Cystine crystals	Pancytopenia	Not described
Pais [7]	F/44 y	Leprosy	Intra-abdominal LN, omentum, and peritoneum	Clofazimine crystals	Mild abdominal pain	Complete remission
Lewis [8]	F/78 y	Eosinophilic colitis	Colon	Charcot–Leyden crystals	Chronic abdominal pain and diarrhea	Not described
Singh [9]	M/19 y	Leprosy	Duodenum and jejunum	Clofazimine crystals	Acute abdominal pain	Complete remission
Szeto [10]	F/68 y	Disseminated *M. abscessus,* clofazimine enteropathy	Terminal ileum	Clofazimine crystals	Abdominal pain, diarrhea, weight loss, and hematochezia	Complete remission
Present case	M/55 y	Strongyloidiasis	Proximal ascending colon	Charcot–Leyden crystals	Epigastric pain	—

F, female; M, male; y, years; SI, small intestine; BM, bone marrow; LN, lymph nodes.
